# Dental Department of Howard University

**Published:** 1894-12

**Authors:** 


THE Dental Department of Howard University of Washington, D. C., has 7 colored and 3 white students.

